# Despite delayed kinetics, people living with HIV achieve equivalent antibody function after SARS-CoV-2 infection or vaccination

**DOI:** 10.3389/fimmu.2023.1231276

**Published:** 2023-08-03

**Authors:** Boitumelo M. Motsoeneng, Nelia P. Manamela, Haajira Kaldine, Prudence Kgagudi, Tandile Hermanus, Frances Ayres, Zanele Makhado, Thandeka Moyo-Gwete, Mieke A. van der Mescht, Fareed Abdullah, Michael T. Boswell, Veronica Ueckermann, Theresa M. Rossouw, Shabir A. Madhi, Penny L. Moore, Simone I. Richardson

**Affiliations:** ^1^ South African Medical Research Council Antibody Immunity Research Unit, Faculty of Health Sciences, University of the Witwatersrand, Johannesburg, South Africa; ^2^ HIV Virology Section, Centre for HIV and STIs, National Institute for Communicable Diseases of the National Health Laboratory Services, Johannesburg, South Africa; ^3^ Department of Immunology, Faculty of Health Science, University of Pretoria, Pretoria, South Africa; ^4^ Division for Infectious Diseases, Department of Internal Medicine, Steve Biko Academic Hospital and University of Pretoria, Pretoria, South Africa; ^5^ South African Medical Research Council Office of AIDS and TB Research, Pretoria, South Africa; ^6^ South African Medical Research Council Vaccines and Infectious Diseases Analytics Research Unit, Faculty of Health Sciences, University of the Witwatersrand, Johannesburg, South Africa; ^7^ African Leadership in Vaccinology Expertise, Faculty of Health Sciences, University of the Witwatersrand, Johannesburg, South Africa; ^8^ Infectious Diseases and Oncology Research Institute, Faculty of Health Sciences, University of the Witwatersrand, Johannesburg, South Africa; ^9^ Centre for the AIDS Programme of Research in South Africa (CAPRISA), University of KwaZulu Natal, Durban, South Africa

**Keywords:** humoral response kinetics, people living with HIV (PLWH), SARS-CoV-2 infection, ChAdOx1 nCov-19 vaccination, neutralization, Fc effector functions

## Abstract

The kinetics of Fc-mediated functions following SARS-CoV-2 infection or vaccination in people living with HIV (PLWH) are not known. We compared SARS-CoV-2 spike-specific Fc functions, binding, and neutralization in PLWH and people without HIV (PWOH) during acute infection (without prior vaccination) with either the D614G or Beta variants of SARS-CoV-2, or vaccination with ChAdOx1 nCoV-19. Antiretroviral treatment (ART)–naïve PLWH had significantly lower levels of IgG binding, neutralization, and antibody-dependent cellular phagocytosis (ADCP) compared with PLWH on ART. The magnitude of antibody-dependent cellular cytotoxicity (ADCC), complement deposition (ADCD), and cellular trogocytosis (ADCT) was differentially triggered by D614G and Beta. The kinetics of spike IgG-binding antibodies, ADCC, and ADCD were similar, irrespective of the infecting variant between PWOH and PLWH overall. However, compared with PWOH, PLWH infected with D614G had delayed neutralization and ADCP. Furthermore, Beta infection resulted in delayed ADCT, regardless of HIV status. Despite these delays, we observed improved coordination between binding and neutralizing responses and Fc functions in PLWH. In contrast to D614G infection, binding responses in PLWH following ChAdOx-1 nCoV-19 vaccination were delayed, while neutralization and ADCP had similar timing of onset, but lower magnitude, and ADCC was significantly higher than in PWOH. Overall, despite delayed and differential kinetics, PLWH on ART develop comparable responses to PWOH, supporting the prioritization of ART rollout and SARS-CoV-2 vaccination in PLWH.

## 1 Introduction

There are more than 38 million people living with HIV (PLWH) globally, two-thirds of whom reside in sub-Saharan Africa (1, 2). South Africa bears the burden of the HIV pandemic with over 7.5 million PLWH, with approximately 2 million not on antiretroviral treatment (ART) (2, 3). Furthermore, PLWH have a 3.2-fold higher probability of testing positive for SARS-CoV-2 and an increased risk for severe COVID-19 and mortality (4–10). PLWH may also be at risk of being reservoirs for the emergence of SARS-CoV-2 variants of concern (VOCs), due to their decreased ability to clear the virus (11–14). Understanding immune responses to SARS-CoV-2 infection and vaccination in PLWH is therefore important for informing vaccine implementation in this population. Binding and neutralizing responses against SARS-CoV-2 infection are reduced in HIV viremic individuals; however, in virally suppressed PLWH, comparable and durable humoral and SARS-CoV-2-specific T-cell responses occur (15–22). Furthermore, COVID-19 severity impacts on the antibody response in PLWH, with severe disease resulting in similar responses and symptomatic non-hospitalized disease in lower magnitude responses compared with people without HIV (PWOH), respectively (23). The BNT162b2, mRNA-1273, Ad26.COV2.S, and ChAdOx1 nCoV-19 vaccines all result in similar binding and neutralizing responses between PLWH and PWOH (17, 24–31). As with infection, stronger vaccine-induced immune responses develop in virally controlled PLWH on ART, compared with ART-naïve PLWH (17, 32–36). However, ART does not fully restore all HIV-specific immune function in PLWH, and additional vaccinations may be required for this at-risk population (37–43).

Beyond neutralization, antibodies mediate cytotoxic functions through the interaction of the antibody Fc region with cellular Fc receptors or complement proteins. These Fc effector functions include antibody-dependent cellular cytotoxicity (ADCC), cellular phagocytosis (ADCP), cellular trogocytosis (ADCT), and complement deposition (ADCD). SARS-CoV-2–specific Fc effector functions have been associated with reduced COVID-19 mortality, are differentially imprinted by SARS-CoV-2 VOCs, are more durable than neutralization activity, and are required for optimal protection conferred by monoclonal antibodies (44–51). Furthermore, Fc effector functions elicited in non-human primates after vaccination correlate with protection (52–57). Apart from a recent study showing similar response rates and levels of ADCP in PLWH and PWOH after infection, the kinetics and longevity of SARS-CoV-2–specific Fc effector function in PLWH are not known (23).

In this study, we investigated SARS-CoV-2 spike-specific Fc functions in hospitalized SARS-CoV-2 vaccine naïve PLWH and PWOH following infection with either D614G or Beta during the acute phase and compared these with ChAdOx1 nCoV-19 vaccinees. ART-naïve PLWH had impaired humoral responses after SARS-CoV-2 infection. Furthermore, subtle differences in Fc-mediated response level and kinetics depending on the infecting variant were observed, and both ADCP and neutralization responses were delayed in PLWH. In comparison with infection, ChAdOx1 nCoV-19 vaccination differed by eliciting delayed binding and higher ADCC in PLWH. Overall, despite early delays in some antibody functions following infection or vaccination, PLWH were able to elicit high levels of humoral immune responses similar to PWOH.

## 2 Materials and methods

### 2.1 Study design

D614G and Beta infection convalescent plasma samples were collected from participants enrolled to COVID-19 study cohorts in South Africa. HIV status was tested prior to enrollment, as part of standard of care. The D614G infection samples (39 PWOH and 14 PLWH) were collected during the months of April to September 2020 prior to the emergence of Beta. The Beta infection samples (13 PWOH and 9 PLWH) were collected between October 2020 and May 2021, when Beta accounted for > 90% of infections in the country (58). Participants were admitted to Steve Biko Academic Hospital (Pretoria, South Africa) with mild to moderate infection (WHO scale 3–5). All samples were collected during the acute phase of COVID-19. Patients had PCR confirmed SARS-CoV-2 infection before blood collection, which was done at admission. A second sample was collected a median of 7 days after admission from participants (D614G, *n* = 37 and Beta, *n* = 14) that were not discharged early or deceased (D614G, *n* = 8 and Beta, *n* = 1). Full demographic and clinical characteristics of the Pretoria COVID-19 study participants are summarized in **Supplementary Table S1**. Ethics approval was received from the University of Pretoria, Faculty of Health Sciences’ Research Ethics Committee (247/2020).

For the ChAdOx1 nCoV-19 vaccine samples, eligible participants were adults living with (*n* = 13) or without HIV (*n* = 17) (**Supplementary Table S2**). Lack of prior infection in these individuals was confirmed by nucleocapsid enzyme-linked immunosorbent assay (ELISA) as previously described (59). Longitudinal sampling was conducted, and plasma was collected 28 days after the 1st dose, at day 42 (14 days after the 2nd dose) and then at day 182 (6 months post-vaccination). The cohort was established at the Chris Hani Baragwanath Hospital as part of the South African safety and efficacy ChAdOx1 nCov-19 randomized trial (28). This study was approved by the Human Research Ethics Committee of the University of the Witwatersrand (ethics reference number: M200501). Written informed consent was obtained from all participants.

### 2.2 Spike plasmid and antigen production

The SARS-CoV-2 Wuhan-1 spike was mutated to include D614G or lineage defining mutations for Beta (L18F, D80A, D215G, 242-244 del, K417N, E484K, N501Y, D614G, and A701V).

For ELISA and Fc effector function assays, SARS-CoV-2 D614G and Beta variant full spike proteins were expressed in human embryo kidney (HEK) 293F suspension cells by transfecting the cells with the respective expression spike plasmids. The cells were incubated for a period of 6 days at 37°C, 70% humidity and 10% CO_2_. Proteins were purified using a nickel-charged resin followed by size-exclusion chromatography. The relevant fractions were collected and stored at −80°C until use.

### 2.3 Cell lines

HEK293F suspension cells were cultured in 293 Freestyle media (Gibco BRL Life Technologies, Ontario, CA) and incubated shaking at 37°C, 5% CO_2_ and 70% humidity at 125 rpm. HEK293T cells were cultured at 37°C, 5% CO_2_, in DMEM (Gibco BRL Life Technologies, Ontario, CA) containing 10% heat-inactivated fetal bovine serum (FBS) and supplemented with 50 μg/ml Gentamicin. Cells were disrupted at confluence with 0.25% trypsin in 1 mM EDTA every 48h–72h. HEK293T/ACE2.MF cells were maintained as for HEK293T cells but were supplemented with 3 μg/ml Puromycin for selection of stably transduced cells. THP-1 cells were used for the ADCP assay and obtained from the AIDS Reagent Program, Division of AIDS, NIAID, NIH contributed by Dr. Li Wu and Vineet N. Kewal Ramani. Cells were cultured at 37°C, 5% CO_2_ in RPMI (Gibco BRL Life Technologies, Ontario, CA) containing 10% heat-inactivated FBS with 1% Penicillin-Streptomycin (Pen/Strep) and 2-mercaptoethanol to a final concentration of 0.05 mM and not allowed to exceed 4 × 10^5^ cells/ml to prevent differentiation. Jurkat-Lucia™ NFAT-CD16 cells (Invivogen, USA) were maintained in IMDM (Gibco BRL Life Technologies, Ontario, CA) media with 10% heat-inactivated FBS, 1% Pen/Strep, and 10 μg/ml of Blasticidin and 100 μg/ml of Zeocin were added to the growth medium every second passage to allow the selection of CD16 expressing cells.

### 2.4 SARS-CoV-2 spike enzyme-linked immunosorbent assay

Two microgram per mililiter of spike protein (D614G or Beta) was used to coat 96-well, high-binding plates and incubated overnight at 4°C. The plates were incubated in a blocking buffer consisting of 5% skimmed milk powder, 0.05% Tween 20, 1× phosphate buffered saline (PBS). Plasma samples were diluted to 1:100 starting dilution in a blocking buffer and added to the plates. IgG secondary antibody was diluted to 1:3000 in blocking buffer and added to the plates followed by TMB substrate (Thermo Fisher Scientific, USA). Upon stopping the reaction with 1 M H_2_SO_4_, absorbance was measured at a 450-nm wavelength. In all instances, mAbs CR3022 and BD23 (a differential control for Beta) were used as positive controls and Palivizumab was used as a negative control.

### 2.5 Lentiviral pseudovirus production

Pseudoviruses were prepared as previously described (60). Briefly, pseudotyped lentiviruses were prepared by co-transfecting the HEK293T cell line with the SARS-CoV-2 variant spike D614G or the Beta spike (L18F, D80A, D215G, 242-244 del, K417N, E484K, N501Y, D614G, and A701V) plasmids in conjunction with a firefly luciferase encoding lentivirus backbone (HIV-1 pNL4.luc) plasmid for 72h. Culture supernatants were clarified of cells by a 0.45 μM filter and stored at −70°C. Other pcDNA plasmids were used for the ADCT assay.

### 2.6 Pseudovirus neutralization assay

For all assays, including the neutralization assay, plasma samples were heat inactivated and clarified by centrifugation. IgG was isolated from the plasma samples of PLWH on ART, using Protein G (Pierce Biotechnology, USA), according to the manufacturer’s instructions and confirmed by IgG ELISA. The plasma samples or IgG preparations from study participants were incubated with the SARS-CoV-2 pseudotyped virus for 1h at 37°C and 5% CO_2_. These samples were also tested against murine leukaemia virus pseudovirus to ensure that there was no interference from ART. Subsequently, 1 × 10^4^ HEK293T cells engineered to overexpress ACE-2 (293T/ACE2.MF) [kindly provided by M. Farzan (Scripps Research)] were added and incubated at 37°C and 5% CO_2_ for 72h upon which the luminescence of the luciferase gene was measured. Titers were calculated as the reciprocal plasma dilution (ID_50_) causing 50% reduction of relative light units (RLUs). CB6 and CA1 were used as neutralization controls for D614G and Beta.

### 2.7 Antibody-dependent cellular phagocytosis (ADCP) assay

SARS-CoV-2 full spike proteins were biotinylated using the EZ-Link Sulfo-NHS-LC-Biotin kit (Thermo Scientific, USA) and coated onto fluorescent neutravidin beads as previously described (61). Briefly, beads were incubated for 2h with monoclonal antibodies at a starting concentration of 20 μg/ml or plasma at a single 1 in 100 dilution. Opsonized beads were incubated with the monocytic THP-1 cell line overnight, fixed and interrogated on the FACSAria II. Phagocytosis score was calculated as the percentage of THP-1 cells that engulfed fluorescent beads multiplied by the geometric mean fluorescence intensity of the population less the no antibody control. For this and all subsequent Fc effector assays, pooled plasma from five PCR-confirmed SARS-CoV-2–infected individuals and CR3022 were used as positive controls and plasma from five pre-pandemic healthy controls and Palivizumab were used as negative controls. 084-7D, a mAb isolated from a donor following SARS-CoV-2 infection, was used as a positive control for Beta. ADCP scores for different spikes were normalized to each other and between runs using CR3022.

### 2.8 FcγRIIIa reporter assay

The ability of plasma antibodies to cross-link spike SARS-CoV-2 protein and activate FcγRIIIa on Jurkat-Lucia™ NFAT-CD16 cells (Invivogen, USA) was measured as a proxy for ADCC. High-binding 96-well plates were coated with 1 μg/ml spike SARS-CoV-2 protein and incubated at 4°C overnight. Plates were then washed with PBS and blocked at room temperature for 1h with 2.5% bovine serum albumin (BSA)/PBS. After washing, heat-inactivated plasma (1:100 final dilution) or monoclonal antibodies (final concentration of 100 μg/ml) in RPMI media supplemented with 10% FBS 1% Pen/Strep were added to the wells and incubated for 1h at 37°C. Jurkat-Lucia™ NFAT- CD16 2 × 10^5^ cells per well were added and incubated for 24h at 37°C and 5% CO_2_. Twenty microliter of supernatant was then transferred to a white 96-well plate with 50 μl of reconstituted QUANTI-Luc secreted luciferase and read immediately on a Victor 3 luminometer with 1 s integration time. The RLUs of a no antibody control were subtracted as background. Palivizumab was used as a negative control, while CR3022 was used as a positive control, and P2B-2F6 to differentiate the Beta from the D614G variant. To induce the transgene, 1× cell stimulation cocktail (Thermo Fisher Scientific, Oslo, Norway) and 2 μg/ml ionomycin in R10 was added. RLUs for spikes were normalized to each other and between runs using CR3022.

### 2.9 Antibody-dependent complement deposition (ADCD) assay

ADCD was measured as previously described (62). Biotinylated spike protein was coated 1:1 onto red fluorescent 1 mM neutravidin beads (Molecular Probes Inc., USA) for 2h at 37°C. These were incubated with a single 1:10 plasma sample dilution or fivefold titration of mAb at a starting concentration of 100 μg/ml for 2h and guinea pig complement diluted 1 in 50 with gelatin/veronal buffer for 15 min at 37°C. Beads were washed in PBS and stained with anti-guinea pig C3b-FITC, fixed and interrogated on a FACSAria II. Complement deposition score was calculated as the percentage of C3b-FITC–positive beads multiplied by the geometric mean fluorescent intensity of FITC in this population less the no antibody or heat-inactivated controls. ADCD scores for D614G and Beta spikes were normalized to each other and between runs using CR3022.

### 2.10 Antibody-dependent cellular trogocytosis (ADCT) assay

ADCT was performed as described in and modified from a previously described study (63). HEK293T cells transfected with a SARS-CoV-2–spike pcDNA vector as above were surface biotinylated with EZ-Link Sulfo-NHS-LC-Biotin as recommended by the manufacturer. Fifty-thousand cells per well were incubated with fivefold titration of mAb starting at 25 μg/ml or single 1 in 100 dilutions for 30 min. Following an RPMI media wash, these were then incubated with carboxyfluorescein succinimidyl ester (CFSE)–stained THP-1 cells (5 × 10^4^ cells per well) for 1h and washed with 15 mM EDTA/PBS followed by PBS. Cells were then stained for biotin using Streptavidin-PE and read on a FACSAria II. Trogocytosis score was determined as the proportion of CFSE-positive THP-1 cells also positive for streptavidin-PE less the no antibody control with infecting variants run head to head.

### 2.11 Dimeric Fc gamma receptor-binding ELISAs

High-binding 96-well ELISA plates were coated with 1 µg/ml spike protein in PBS overnight at 4°C. Three wells on each plate were directly coated with 5 µg/ml IgG, isolated from healthy donors, and signals from these wells were used to normalize the Fc receptor activity of the plasma samples. Plates were washed with PBS and blocked with PBS/1 mM EDTA/1% BSA for 1h at 37°C. Plates were then washed and incubated with 1:10 diluted plasma for 1h at 37°C and then with 0.2 µg/ml or 0.1 µg/ml of biotinylated FcγRIIa or FcγRIIIa dimer, respectively (constructs kindly provided by Prof. Mark Hogarth from the Burnet Institute, Australia), for 1h at 37°C (64). Subsequently, a 1:10000 dilution of Pierce high-sensitivity streptavidin-horseradish peroxidase (Thermo Scientific, USA) was added for a final incubation for 1h at 37°C. Last, TMB substrate (Sigma-Aldrich) was added, and color development was stopped with 1 M H_2_SO_4_ and absorbance read at 450 nm.

### 2.12 Quantification and statistical analysis

Analyses were performed in Prism (v9; GraphPad Software Inc, San Diego, CA, USA). Non-parametric tests were used for all comparisons. Fisher’s exact test was used to compare categorical variables between the study participants living with or without HIV, when assessing the clinical data. The Mann-Whitney test was used for unmatched samples. The Kruskal-Wallis test with Dunn’s correction was conducted for multiple comparisons. All correlations reported are non-parametric Spearman’s correlations. *P*-values less than 0.05 were considered to be statistically significant.

## 3 Results

### 3.1 People living with HIV have similar levels of humoral responses irrespective of the SARS-CoV2 infecting variant

We first compared antibody responses in SARS-CoV-2–infected PLWH with PWOH and assessed whether the infecting variant differentially triggered antibody functions. We used plasma from patients hospitalized during South Africa’s first (PWOH, *n* = 39 and PLWH, *n* = 14) and second (PWOH, *n* = 13 and PLWH, *n* = 9) SARS-CoV-2 infection waves, which were dominated by the D614G and Beta variants, respectively. PWOH and PLWH were matched for age, sex assigned at birth, comorbidities, and SARS-CoV-2 disease severity for each of the infecting variants (**Supplementary Table S1**). Samples were collected at admission, which was a median of 2 days after a positive SARS-CoV-2 PCR test. During the acute infection, a second sample was taken a median of 7 days later, for a subset of patients who had not been discharged or were not deceased by this time (**Supplementary Table S1**).

**Figure 1:**
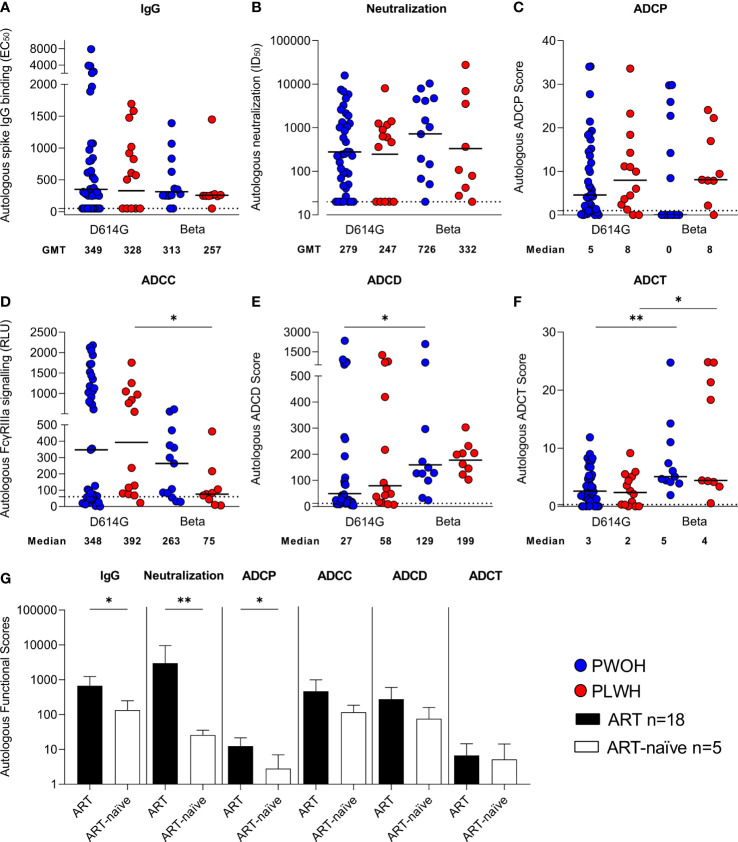
Similar antibody responses in hospitalized patients living with or without HIV during SARS-CoV-2 D614G and Beta infections. Autologous antibody responses at admission against D614G spike for D614G infections and Beta spike for Beta infections in people without HIV (PWOH) (D614G, *n* = 39; Beta, *n* = 13) are shown in blue and for people living with HIV (PLWH) (D614G, *n* = 14; Beta, *n* = 9) are shown in red. **(A)** IgG binding by ELISA against autologous full-length spikes. **(B)** Neutralization of D614G or Beta pseudoviruses. **(C)** Antibody-dependent cellular phagocytosis (ADCP) scores, as the percentage of monocytic cells that take up spike-coated beads multiplied by their geometric mean fluorescence intensity (MFI). **(D)** ADCC shown as relative light units (RLUs) signaling through FcγRIIIa-expressing cells. **(E)** ADCD is shown as the MFI of C3b deposition on spike-coated beads and **(F)** ADCT represented as the relative proportion of biotinylated spike-expressing cell membrane on carboxyfluorescein succinimidyl ester (CFSE)–positive monocytic cells. The lines represent geometric mean titers for the IgG and neutralization responses, and the median for the Fc-mediated responses. Limits of detection are shown with dotted lines. Kruskal-Wallis test with Dunn’s correction was calculated for multiple test comparisons. **(G)** Autologous functional scores, referring to the activity of either the IgG-binding EC_50_, neutralization ID_50_, ADCP score, FcγRIIIa signaling RLU, ADCD score, or ADCT score measured against spike protein matching the infecting variant, among PLWH on antiretroviral treatment (ART) or ART-naïve at admission. PLWH on ART (*n* = 18), data shown as filled bars and ART-naïve participants (*n* = 5), data shown as unfilled bars. Error bars indicate standard deviation of the mean of participant data. Mann Whitney U test was used to compare groups. Statistical significance: **p < 0.01; *p < 0.05.

SARS-CoV-2 IgG autologous spike binding (binding to the D614G spike for D614G infections and Beta spike for Beta infections) was measured at admission using an ELISA. The magnitude of IgG antibodies was similar irrespective of infecting variant and by HIV status [D614G geometric mean titers (GMTs) PWOH 349 vs. PLWH 328 and Beta GMTs PWOH 313 vs. PLWH 257] (**Figure 1A**). There were also no significant differences in pseudovirus neutralization titers by HIV status or across the infecting variants of SARS-CoV-2 infection (D614G GMTs PWOH 279 vs. PLWH 247 and Beta GMTs PWOH 726 vs. PLWH 332) (**Figure 1B**). SARS-CoV-2 spike-specific Fc effector responses were similar among the patients regardless of their HIV status (**Figures 1C–F**). While ADCP was not different across the infecting variants (**Figure 1C**), the Beta variant induced lower ADCC activity in PLWH (medians Beta 75 vs. D614G 392 *p* = 0.037) (**Figure 1D**), higher ADCD in PWOH (medians Beta 129 vs. D614G 27 *p* = 0.024) (**Figure 1E**) and higher levels of ADCT in both PWOH (medians Beta 5 vs. D614G 3 *p* = 0.007) and PLWH (medians Beta 4 vs. D614G 2 *p* = 0.022) (**Figure 1F**). Overall, PLWH had similar functional responses to PWOH during infection by either variant. However, the Beta variant differentially induced Fc-mediated functions similar to our previous findings (44).

As Fc effector function is modulated by Fc receptor binding, we examined the ability of antibodies from D614G and Beta infections to cross-link dimeric FcγRIIa or FcγRIIIa receptors (which modulate ADCP and ADCC, respectively) and the D614G or Beta spike protein by ELISA. As expected, Spearman’s correlations > 0.5 *p* < 0.001 were noted between FcγRIIa binding and ADCP score, and between FcγRIIIa binding and ADCC reporter assay against D614G spike (**Supplementary Figures S1A, B**). Similar to the functional readouts, there were no significant differences in FcγRIIa binding (**Supplementary Figure S1C**) by HIV status. For the Beta infections, we observed lower FcγRIIa binding in comparison with the D614G infections (*p* = 0.021). However, no differences were noted between Beta- and D614G-triggered ADCP (**Figure 1C**), suggesting the potential impact of variation in antigen presentation on functional bead-based and soluble protein assays. The FcγRIIIa cross-linking (**Supplementary Figure S1D**) and ADCC reporter assay, where FcγRIIIa is expressed on a target cell, both showed similar responses in hospitalized patients, regardless of HIV status, but the Beta variant compared with D614G resulted in significantly reduced FcγRIIIa binding (*p* = 0.010).

### 3.2 SARS-CoV-2 humoral responses are compromised in people living with HIV not on antiretroviral treatment

PLWH were stratified according to whether they were on ART (*n* = 18, all of whom had CD4 T-cell counts greater than 100 cells/µl) or ART-naïve (*n* = 5, all with CD4 T-cell counts lower than 100 cells/µl). The ART-naïve PLWH had significantly lower IgG binding (*p* = 0.037), neutralization (*p* = 0.006), and ADCP (*p* = 0.012) activity than the PLWH on ART (**Figure 1G**). Additionally, both ADCC and ADCD responses were lower in ART-naïve individuals, although not significantly so, while ADCT responses were comparable between the two groups. Overall, this indicates that PLWH who do not receive ART have impaired immune responses to SARS-CoV-2 infection, as has been previously reported (15–18).

### 3.3 People living with HIV have delayed neutralization and ADCP SARS-CoV-2 responses following D614G infection

**Figure 2:**
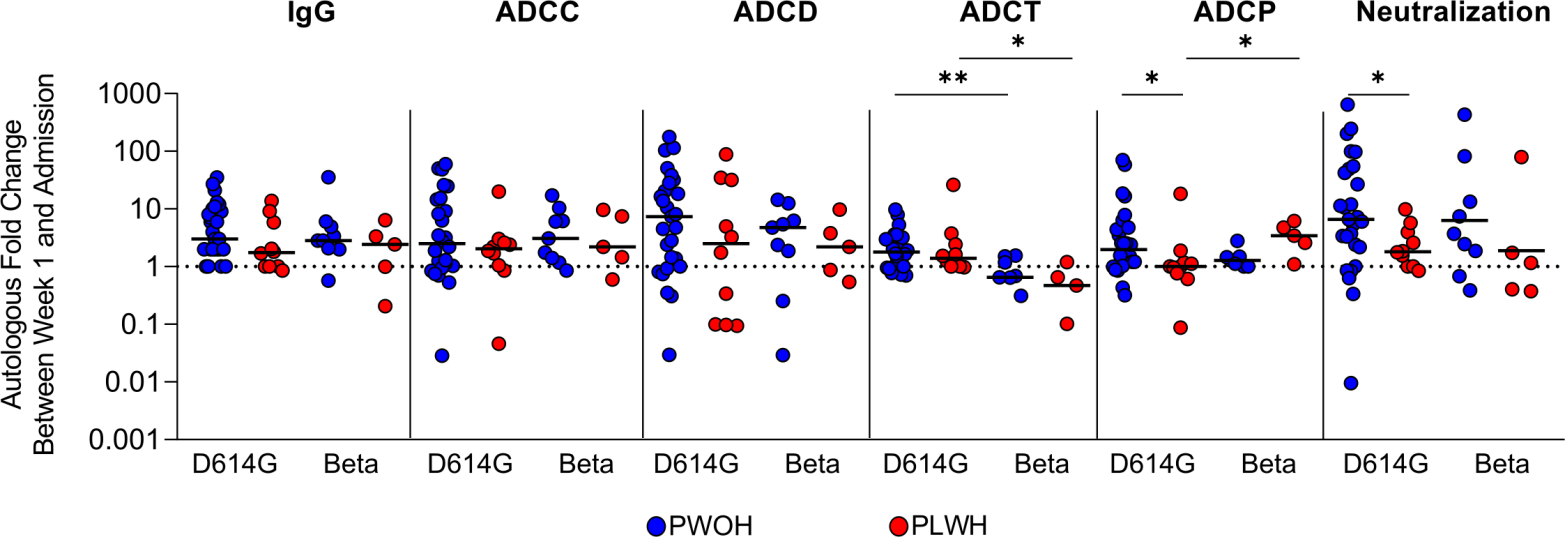
People living with HIV have delayed humoral response kinetics following SARS-CoV-2 D614G infection. Fold changes in autologous antibody responses a week after admission during SARS-CoV-2 D614G or Beta infection in PWOH (D614G, *n* = 27; Beta, *n* = 9) shown in blue and for PLWH (D614G, *n* = 10; Beta, *n* = 5) shown in red. The dotted line indicates no change between sampling time points, above 1 indicates an increase in function and below 1 indicates a decrease in function. The median of the fold changes is indicated by solid lines. For each of the antibody responses Kruskal-Wallis test with Dunn’s correction was calculated for multiple test comparison. Statistical significance: **p < 0.01; *p < 0.05.

We next investigated the kinetics of humoral responses between admission and 1 week of hospitalization in PLWH and PWOH following infection with D614G (PWOH, *n* = 27 and PLWH, *n* = 10) or Beta (PWOH, *n* = 9 and PLWH, *n* = 5). This analysis excluded patients that were either discharged early or deceased. Overall, humoral responses increased between admission and 1 week of hospitalization, as indicated by a fold change greater than 1 (week 1/admission responses), regardless of HIV status and the infecting variant (**Figure 2**). There were no significant differences in the median fold changes of IgG binding, ADCC, ADCD, and ADCT by HIV status, indicating that these functional responses increased at the same rate in PLWH as in PWOH. However, during D614G infection, neutralization (*p* = 0.042) and ADCP responses (*p* = 0.032) had significantly lower fold changes in PLWH, indicating that these responses were delayed compared with PWOH. The Beta variant had slower ADCT responses in both PWOH (*p* = 0.001) and PLWH (*p* = 0.021), and in contrast to D614G, Beta rapidly elicited ADCP in PLWH (**Figure 2**). These data suggest that some humoral responses are delayed in PLWH following acute SARS-CoV-2 infection and that different variants elicit altered levels of activity and also affect the kinetics of humoral responses.

### 3.4 People living with HIV show stronger coordination between SARS-CoV-2 IgG binding, neutralization, and Fc effector functions following infection

**Figure 3:**
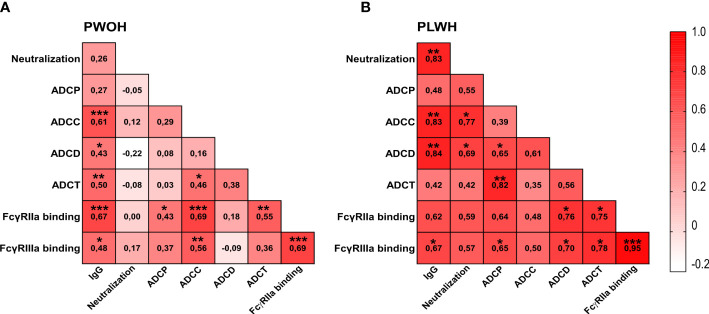
Differential associations between antibody responses in hospitalized patients living with or without HIV. Spearman correlations for **(A)** PWOH (*n* = 27) and **(B)** PLWH (*n* = 10) 1-week post-admission during SARS-CoV-2 D614G infection. Color intensity from white to red is associated with increasing correlation, indicated within the blocks. Statistical significance: ***p < 0.001; **p < 0.01; *p < 0.05.

A coordinated antibody response against other diseases has been shown following vaccination and for HIV associated with improved protection (53, 65–67). We therefore assessed the correlations between antibody functions in PLWH (*n* = 10) and PWOH (*n* = 27) 1 week after admission following D614G infection. In PWOH, the strongest significant associations (*r* > 0.5 *p* < 0.05) with IgG binding were ADCC (*r* = 0.61 *p* < 0.001), ADCT (*r* = 0.50 *p* < 0.01), and FcγRIIa binding (*r* = 0.67 *p* < 0.001) (**Figure 3A**). In PLWH, SARS-CoV-2 spike IgG binding had a strong significant association with neutralization (*r* = 0.83 *p* < 0.01), ADCC (*r* = 0.83 *p* < 0.01), ADCD (*r* = 0.84 *p* < 0.01), and FcγRIIIa binding (*r* = 0.67 *p* < 0.05) (**Figure 3B**). The most striking difference between the groups was that neutralization activity was significantly associated with ADCC (*r* = 0.77 *p* < 0.05) and ADCD (*r* = 0.69 *p* < 0.05) in PLWH, but there were no significant associations between the neutralization activity and other functions in PWOH. Similarly, ADCP strongly correlated with ADCD (*r* = 0.65 *p* < 0.05), ADCT (*r* = 0.82 *p* < 0.01), and FcγRIIIa binding (*r* = 0.65 *p* < 0.05), and both ADCD and ADCT correlated with FcγRIIa (*r* = 0.76 *p* < 0.05 and *r* = 0.75 *p* < 0.05 respectively) and FcγRIIIa binding (*r* = 0.7 *p* < 0.05 and *r* = 0.78 *p* < 0.05, respectively) in PLWH. In comparison with the Fc-mediated functions of PLWH, fewer significant associations were observed in PWOH. Strong correlations were only observed for ADCC with FcγRIIa (*r* = 0.69 *p* < 0.001) and FcγRIIIa binding (*r* = 0.56 *p* < 0.01), and ADCT with FcγRIIa binding (*r* = 0.55 *p* < 0.01). Thus, antibody functional relationships differ between PLWH and PWOH, and PLWH in this study had a more coordinated functional response to SARS-CoV-2 infection, despite slower neutralization and ADCP kinetics in D614G infection.

### 3.5 ChAdOx1 nCoV-19 vaccination elicits higher IgG binding and ADCC over time in people living with HIV

**Figure 4:**
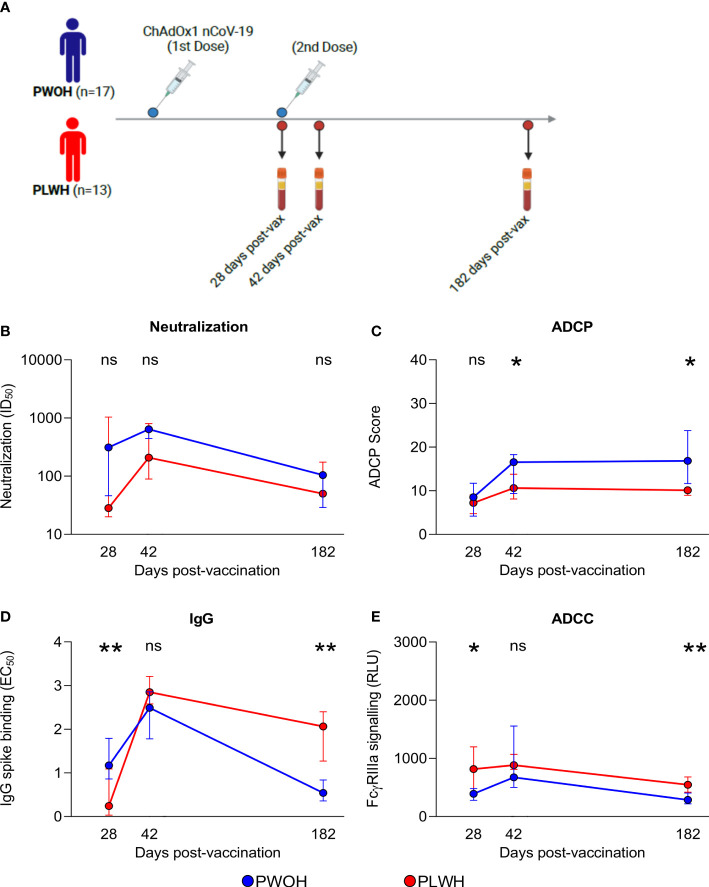
People living with HIV have durable humoral responses following ChAdOx1 nCoV-19 vaccination. **(A)** Thirty participants received two doses of the ChAdOx1 nCoV-19 vaccine, antibody responses were assessed for days 28, 42, and 182 post-vaccinations when plasma samples were collected from PWOH (*n* = 17) shown in blue and PLWH (*n* = 13) shown in red. **(B)** Neutralization of D614G pseudovirus, **(C)** ADCP scores, **(D)** IgG binding by ELISA, and **(E)** ADCC FcγRIIIa signalling were measured. The lines represent the medians with error bars, indicating standard deviation of participant data. Mann Whitney U test was calculated to compare groups at each of the time points. Statistical significance: **p < 0.01; *p < 0.05; ns, not significant.

The initial SARS-CoV-2 vaccines were based on the ancestral spike sequence, enabling us to examine IgG binding, neutralization, and Fc effector functions following ChAdOx1 nCoV-19 vaccination, to determine whether the kinetics are similar to D614G infection responses in both PLWH and PWOH. Thirty participants stratified by HIV status (PWOH, *n* = 17 and PLWH, *n* = 13) received two doses of the vaccine and plasma samples were collected at days 28 (after first dose), 42 (after second dose) and 182 post-vaccination (**Figure 4A**). The PWOH and PLWH were matched for age, but PLWH had a lower proportion of males (**Supplementary Table S2**). Among the PLWH, the majority (77%) were on ART and had CD4 T-cell counts greater than 250 cells/µl. The onset of neutralization responses was similar regardless of HIV status, although this functional response trended lower in PLWH (**Figure 4B**). At 28 days post-vaccination, the onset of ADCP responses was similar between the groups, but the maturation of ADCP following the second dose in PLWH was slowed, resulting in significantly lower activity at days 42 (*p* = 0.038) and 182 (*p* < 0.011) (**Figure 4C**).

In contrast to infection, IgG binding was the only response significantly delayed at day 28 (*p* = 0.003) in PLWH. However, following the second dose, PLWH achieved comparable levels and then exceeded the titers observed in PWOH at 182 days (*p* = 0.002) (**Figure 4D**). In comparison with the other functions, ADCC was rapidly elicited in PLWH with significantly higher levels at day 28 (*p* = 0.035) than in the PWOH, and following the second dose, these higher levels persisted through to day 182 (*p* = 0.005) (**Figure 4E**). Last, we observed similar antibody kinetics between ART-treated and ART-naïve PLWH, likely due to small participant numbers, following ChAdOx1 nCoV-19 vaccination (data not shown). In contrast to infection with D614G, there was an initial delay in IgG binding, which may have compromised the levels and kinetics of neutralization and ADCP responses in PLWH, although not significantly. Despite early delays in antibody functions, ChAdOx1 nCoV-19 vaccination was able to induce higher IgG binding and ADCC in PLWH than in PWOH.

## 4 Discussion

PLWH are at high risk of COVID-19 morbidity and mortality (5–10), therefore investigating the kinetics of antibody responses is essential for understanding protection against SARS-CoV-2 and informing vaccine implementation. Here, we confirm that ART-naïve individuals showed compromised SARS-CoV-2 immunity. We show that the infecting variants triggered nuanced differences in the levels and kinetics of Fc effector functions, as we have previously reported (44). Neutralization and ADCP responses were significantly delayed in PLWH, but despite these delays, a more coordinated antibody functional response was observed in comparison with PWOH. Following ChAdOx1 nCoV-19 vaccination, PLWH overall had slightly impaired neutralization and ADCP responses, and spike binding was delayed; however, ADCC was significantly higher. This study highlights that the kinetics of Fc-mediated functions differ by SARS-CoV-2 infection and vaccination in PLWH and despite initial delays, PLWH are able to reach an equivalent humoral immune response to PWOH.

Our observation that ART-naïve PLWH (CD4 counts less than 100 cells/µl) had significantly lower IgG binding, neutralization, and ADCP responses suggests impaired SARS-CoV-2 immunity in this immune-compromised population. This corroborates previous studies that showed lower IgG and pseudovirus neutralizing antibody titers in virally unsuppressed PLWH with low CD4 counts between 200 and 500 cells/ul (15–18). In contrast, several studies reported unimpaired binding and neutralization in the context of ART-controlled HIV, who had even higher CD4 counts than participants in our study (19, 20). Most PLWH in our cohort accessed ART, likely explaining why we saw no overall impairment in humoral responses (68–70).

Our study also investigated Fc effector functions ADCC, ADCD, and ADCT, which have not previously been measured in PLWH following SARS-CoV-2 infection. We found no differences in Fc effector functions compared with PWOH. This confirms a previous finding that ADCP in PLWH after SARS-CoV-2 infection showed no difference compared with PWOH (23). Thus, PLWH on ART were capable of eliciting a robust Fc-mediated response after SARS-CoV-2 infection, similar to PWOH.

However, differences in the magnitude of Fc effector functions were observed depending on which infecting variant triggered the response. Beta induced significantly lower ADCC activity in PLWH and also trended lower in PWOH. In PLWH, ADCD trended higher and was shown to be significantly higher in PWOH following Beta infection. These observations were similar to our previous findings in a different cohort of PWOH, in which ADCC was reduced and ADCD was substantially higher in individuals infected with Beta (44). Here, Beta elicited higher levels of ADCT in both PWOH and PLWH, but in our previous study, responses were unaffected in comparison with D614G (44). This difference in studies suggests that other factors, such as disease severity, may also impact variant imprinting. Here, and in our previous study, we conclude that the sequence of infecting variants may alter antibody quality, perhaps by eliciting antibodies with varying glycosylation and/or isotype, a finding which requires further investigation.

While no quantitative differences in the level of responses were noted at enrollment, both neutralization and ADCP were delayed in PLWH following D614G infection. These delays were not observed for PLWH infected with Beta despite similarities in age, CD4 counts, and the proportion of individuals on ART, when compared with PLWH infected with D614G. This indicates that there is an impairment in SARS-CoV-2 responses in PLWH, perhaps due to the incomplete restoration of immune function following ART (37, 38). Delayed responses could increase the risk of re-infections or vaccine breakthrough infections and have been associated with SARS-CoV-2 RNAemia and COVID-19 progression (71). However, these same delays were not noted following ChAdOx1 nCoV-19 vaccination. Vaccinated PLWH rapidly elicited robust ADCC responses exceeding the levels in PWOH after the first dose and these levels remained higher 6 months post-vaccination. ChAdOx1 nCoV-19–induced ADCC in PWOH showed greater cross-reactivity against Delta and lasted longer than binding and neutralizing antibodies as previously reported (72). This highlights that ADCC may also be an important mechanism for durable and broad vaccine-induced immunity in PLWH but requires further investigation into how this functional response is differentially regulated in these individuals. In contrast to comparable binding kinetics observed for D614G infection, binding responses were initially delayed in ChAdOx1 vaccinated PLWH. This could be due to lower antigen exposure in vaccination, as the only difference in spike immunogen sequences is the D614G mutation. However, these binding responses increased to equivalent levels in PWOH after the second vaccine and result in higher binding at 6 months post-vaccination. In an immunogenicity study for the ChAdOx1 vaccine, including larger numbers of PLWH, higher SARS-CoV-2 spike binding antibodies were reported with each vaccine dose in PLWH (28). The durability and higher binding is potentially due to the late onset of this response in PLWH compared with PWOH. While neutralization and ADCP were delayed in infection, after vaccination, these responses had similar kinetics but lower levels in PLWH, although not significantly so for neutralization. Overall, vaccination induces different effects compared with D614G infection despite a similar antibody-targeted immunogen.

In addition to delayed responses following infection, PLWH had a surprisingly better coordination of antibody functional responses. For SARS-CoV-2, early infection that triggers an enhanced coordinated Fc effector response has been associated with COVID-19 recovery (47, 48). Here, our data suggest that PLWH have a unique trajectory in humoral responses against SARS-CoV-2. This is not restricted to SARS-CoV-2 responses, as we have previously reported on a distinct coordination of antibody responses in PLWH in comparison with PWOH after influenza vaccination (66). However, the mechanisms that drive this more coordinated response in PLWH require further investigation. Given that there was no difference in severity or death between PLWH and PWOH in this cohort, it is possible that a more balanced or synergistic immune response, despite delays in their development, may be required for protection in this population.

Limitations of this study include the lack of sequences for the infecting variant; however, this was mitigated by sampling when the circulating variant accounted for > 90% of infections in the population. For Beta-triggered neutralization responses, PWOH had higher responses; however, the lack of statistical significance when compared with responses in PLWH is possibly a result of small participant numbers. In several cases, due to small numbers of ART-naïve PLWH, we pooled data from both waves, limiting our ability to assess the impact of the infecting variant. In addition, HIV viral loads were not available for all participants and, as a result, viral suppression could not be defined. Furthermore, sample sizes for the kinetics and coordination analysis were reduced due to the lack of follow-up samples from some patients. The SARS-CoV-2 infection humoral kinetics reported in this study are restricted to the first week following infection, which corresponds to the acute phase. Therefore, unlike in vaccination where longitudinal samples were obtained, a more complete analysis of the infection humoral kinetics was not possible.

In conclusion, our study shows that SARS-CoV-2 infection induces different kinetics of responses to vaccination between PLWH and PWOH. These data highlight the importance of rapid ART rollout and support the current SARS-CoV-2 vaccine implementation strategies in PLWH. Despite delayed kinetics, PLWH were able to elicit comparable responses to SARS-CoV-2 infection and vaccination. This study highlights the importance of investigating the kinetics of humoral immune responses against SARS-CoV-2 in PLWH, as these provide insights into the mechanisms required for immunity against severe disease and vaccine efficacy in this population.

## Data availability statement

The original contributions presented in the study are included in the article/**Supplementary Material**. Further inquiries can be directed to the corresponding authors.

## Ethics statement

The studies involving human participants were reviewed and approved by University of Pretoria, Faculty of Health Sciences’ Research Ethics Committee (247/2020) and Human Research Ethics Committee of the University of the Witwatersrand (ethics reference number: M200501). The patients/participants provided their written informed consent to participate in this study.

## Author contributions

BM, PM, and SR conceptualized the study. BM performed experiments, analysed data, generated the figures and wrote the manuscript. NM and SR performed Fc experiments and analysed data. HK and PK performed neutralization assays supervised by TH. FAy and ZM performed ELISA assays supervised by TM-G. MM processed samples and FAb, MB, VU, and TR established the Pretoria COVID-19 study, which provided participant samples from the Tshwane District Hospital. SM provided samples and clinical data for the ChAdOx1-nCoV19 vaccination trial. PM and SR assisted in data interpretation, reviewed and edited the manuscript and supervised the research. All authors contributed to the manuscript and approved the submitted version.
