# Supplementary material for: Despite delayed kinetics, people living with HIV achieve equivalent antibody function after SARS-CoV-2 infection or vaccination
Source: Front Immunol. 2023 Aug 3;14:1231276. doi: 10.3389/fimmu.2023.1231276 (PMC10435738; doi:10.3389/fimmu.2023.1231276)
Supplement: Supplementary file 1 [file DataSheet_1.docx]

***Supplementary Material***

**Despite delayed kinetics, people living with HIV achieve equivalent antibody function after SARS-CoV-2 infection or vaccination**

**Boitumelo M. Motsoeneng, Nelia P. Manamela, Haajira Kaldine, Prudence Kgagudi, Tandile Hermanus, Frances Ayres, Zanele Makhado, Thandeka Moyo-Gwete, Mieke A. van der Mescht, Fareed Abdullah, Michael T. Boswell, Veronica Ueckermann, Theresa M. Rossouw, Shabir A. Madhi, Penny L. Moore^*^ and Simone I. Richardson^*^**

***Correspondence:**Penny L. Moore Simone I. Richardson

[pennym@nicd.ac.za](mailto:pennym@nicd.ac.za) [simoner@nicd.ac.za](mailto:simoner@nicd.ac.za)

**Table S1: Demographic and clinical details of the D614G and Beta COVID-19 infection cohorts, stratified by HIV status**

|  | **D614G** | | | **Beta** | | |
| --- | --- | --- | --- | --- | --- | --- |
|  | **PWOH (n=39)** | **PLWH (n=14)** |  | **PWOH (n=13)** | **PLWH (n=9)** |  |
| **Demographics** | | | | | | |
| Median age; years [IQR] | 47 [42-56] | 46 [44-52] | ns | 46 [42-52] | 37 [36-56] | ns |
| Gender Male: Female (% Female) | 24:15 (38%) | **^a^** 4:10 (71%) | ns | 6:7 (54%) | 2:7 (78%) | ns |
| **Clinical** | | | | | | |
| ***Comorbidities; n (%)*** | | | | | | |
| Hypertension | 23 (59%) | 4 (29%) | ns | 5 (38%) | 4 (44%) | ns |
| Diabetes | 16 (41%) | 3 (20%) | ns | 2 (15%) | 1 (11%) | ns |
| Obesity | 21 (54%) | 6 (43%) | ns | 5 (38%) | 3 (33%) | ns |
| History of tuberculosis | 1 (3%) | 2 (13%) | ns | 0 | 0 | ns |
| Chronic kidney disease | 3 (8%) | 0 | ns | 2 (15%) | 0 | ns |
| Cardiovascular disease | 8 (21%) | 2 (13%) | ns | 2 (15%) | 1 (11%) | ns |
| Cancer | 1 (3%) | 0 | ns | 0 | 1 (11%) | ns |
| >1 comorbidity | 24 (62%) | **^b^** 4 (29%) | ns | 6 (46%) | **^b^** 4 (44%) | ns |
| No comorbidities | 7 (18%) | **^c^** 2 (13%) | ns | 5 (38%) | **^c^** 5 (55%) | ns |
| ***HIV infection*** | | | | | | |
| ART, n (%) | n/a | 11 (73%) | n/a | n/a | 7 (78%) | n/a |
| Median CD4+ count; cells/μl [IQR] | n/a | 260 [113-352] | n/a | n/a | 137 [70-514] | n/a |
| ***SARS-CoV-2 infection*** | | | | | | |
| **^d^** PCR positivity | 39 (100%) | 14 (100%) | n/a | 13 (100%) | 9 (100%) | n/a |
| Test to admission; days [IQR] | 2 [1-3] | 2 [0-5] | ns | 1 [0-3] | 0 [0-2] | ns |
| Post-symptom onset at admission; days [IQR] | 8 [7-11] | 9 [6-11] | ns | 7 [4-9] | 5 [3-11] | ns |
| Duration of admission; days [IQR] | 9 [6-13] | 9 [7-14] | ns | 7 [5-9] | 7 [4-8] | ns |
| Inpatients for ≥ 1 week | 27 (69%) | 10 (71%) | ns | 9 (69%) | 5 (56%) | ns |
| ***Disease severity at admission*** | | | | | | |
| **^e^** WHO Scale 3 mild | 5 (13%) | 2 (13%) | ns | 3 (23%) | 4 (44%) | ns |
| **^f^** WHO Scale 4 moderate | 23 (59%) | 10 (73%) | ns | 9 (69%) | 5 (56%) | ns |
| **^g^** WHO Scale 5 moderate | 6 (15%) | 0 | ns | 0 | 0 | ns |
| Deaths | 7 (18%) | 1 (7%) | ns | 0 | 1 (11%) | ns |
| Mann-Whitney test used to compare between the study participants living without or with HIV. Fisher's exact test used to compare categorical variables between the study participants living without or with HIV. | | | | | | |
| **^a^** Pregnant (1) |  |  |  |  |  |  |
| **^b^** More than 1 co-morbidity in addition to HIV | |  |  |  |  |  |
| **^c^** No co-morbidities in addition to HIV |  |  |  |  |  |  |
| **^d^** Variant not sequenced but all tests conducted between April and September 2020 (South Africa’s 1st wave; D614G dominance) and between October 2020 to May 2021 (South Africa’s 2nd wave; Beta dominance) | | | | | | |
| **^e^** Ambulatory mild, symptomatic assistance needed | | | | | | |
| **^f^** Hospitalized moderate, no oxygen therapy | |  |  |  |  |  |
| **^g^** Hospitalized moderate, oxygen by mask or nasal prongs | |  |  |  |  |  |
| Statistical significance: ns=not significant; n/a=not applicable | | | |  |  |  |

**Table S2: Demographic and clinical details of the ChAdOx1 nCoV-19 vaccine trial participants, stratified by HIV status**

|  | **PWOH (n=17)** | **PLWH (n=13)** |  |
| --- | --- | --- | --- |
| **Demographics** |  |  |  |
| Median age; years [IQR] | 31 [24-37] | 35 [31-44] | ns |
| Gender Male: Female (% Female) | 10:7 (41%) | 2:11 (85%) | * |
| **HIV infection** | | | |
| ART, n (%) | n/a | 10 (77%) | n/a |
| Median CD4+ count; cells/μl [IQR] | n/a | 737 [615-901] | n/a |
| Mann-Whitney test used to compare between the study participants living without or with HIV. Fisher's exact test used to compare categorical variables between the study participants living without or with HIV.  Statistical significance: *p <0.05; ns=not significant; n/a=not applicable | | | |


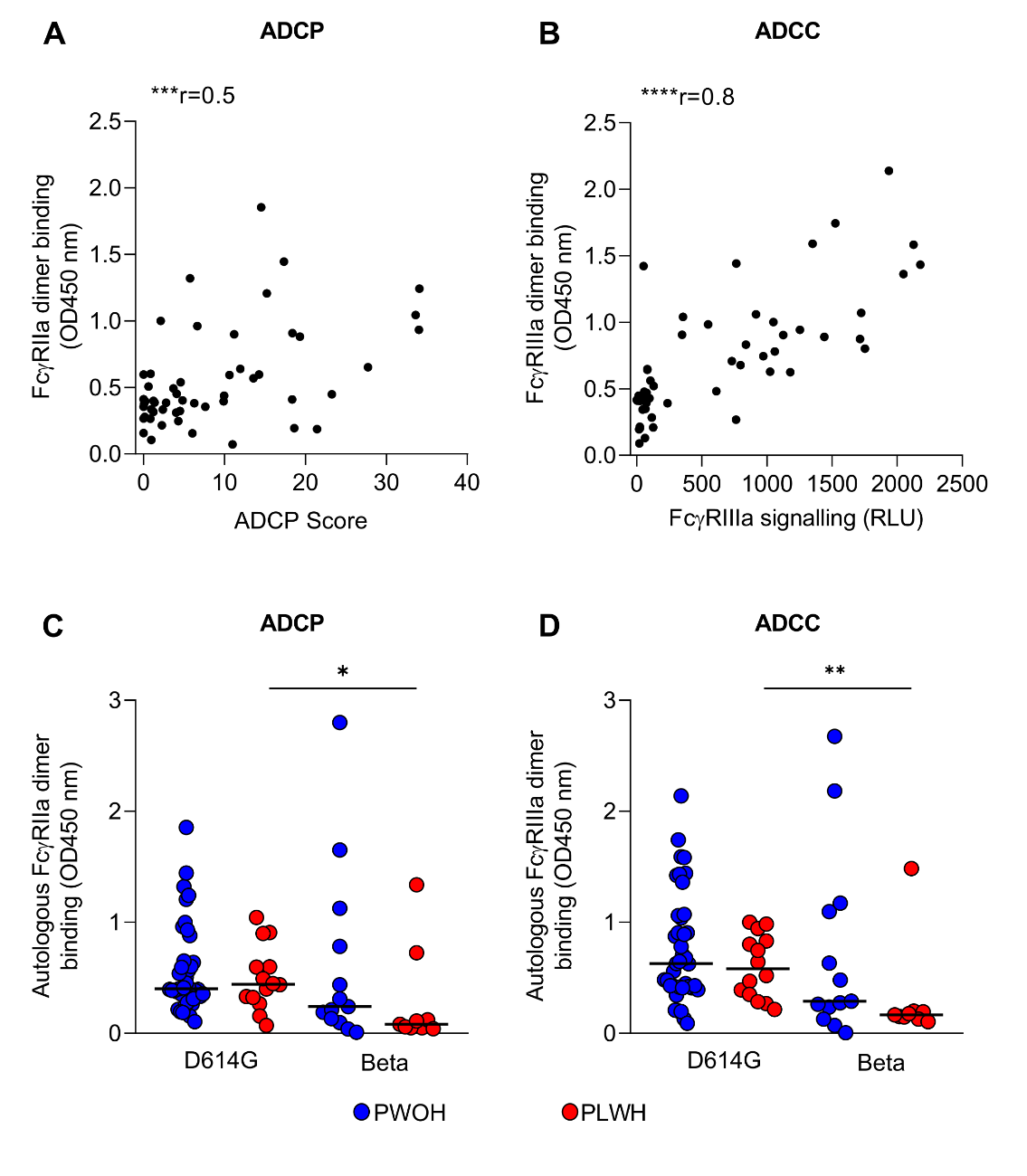


**Supplementary Figure 1. Fc gamma receptor (FcγR) binding correlates with functional activity against SARS-CoV-2**. Spearman’s correlations of **(A)** FcγRIIa dimer binding by ELISA and ADCP score by flow cytometry-based assay and **(B)** FcγRIIIa dimer binding by ELISA and FcγRIIIa signalling relative light units (RLU) by ADCC reporter assay of D614G infection plasma samples (n=53) against D614G spike. **(C)** FcγRIIa and **(D)** FcγRIIIa dimer binding against autologous spikes, D614G and Beta. FcγR dimer binding for PWOH shown in blue (D614G n=39 and Beta n=13) and for PLWH shown in red (D614G n=14 and Beta n=9). The lines represent the median. Kruskal-Wallis test with Dunn’s correction was calculated for multiple test comparisons. Statistical significance: ****p < 0.0001; ***p < 0.001; **p < 0.01; *p < 0.05

**
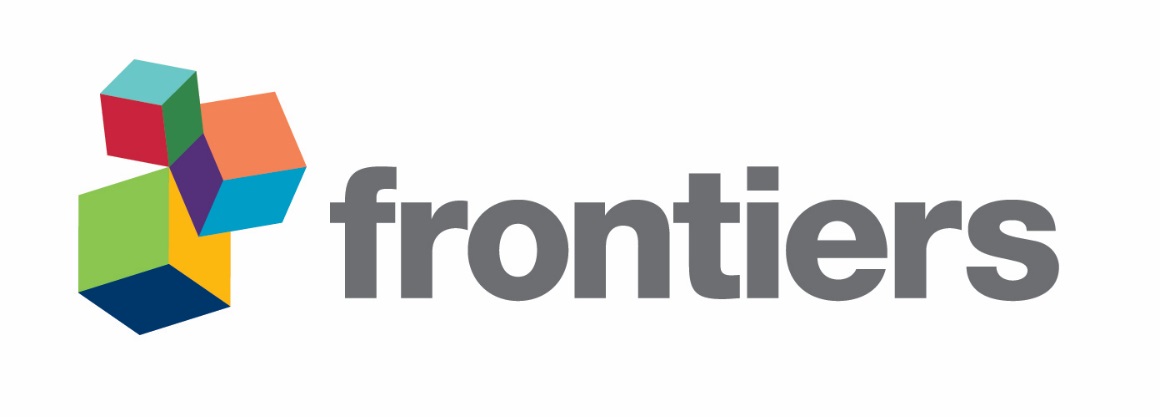
**
